# Wisent Somatic Cells Resist Reprogramming by the PiggyBac Transposon System: A Case Study Highlighting Methodological and Conservation Hurdles

**DOI:** 10.3390/ijms26094327

**Published:** 2025-05-02

**Authors:** Marta Marlena Ziętek, Ajna Bihorac, Elżbieta Wenta-Muchalska, Anna Maria Duszewska, Wanda Olech, Silvestre Sampino, Agnieszka Bernat

**Affiliations:** 1 Department of Experimental Embryology, Institute of Genetics and Animal Biotechnology Polish Academy of Sciences, 05-552 Jastrzębiec, Poland; 2 Department of Morphological Sciences, Faculty of Veterinary Medicine, Warsaw University of Life Sciences, 02-787 Warszawa, Poland; 3 Department of Animal Genetics and Conservation, Faculty of Animal Science, Warsaw University of Life Sciences, 02-787 Warszawa, Poland; 4 Laboratory of Photobiology and Molecular Diagnostics, Intercollegiate Faculty of Biotechnology, University of Gdansk & Medical University of Gdansk, 80-307 Gdansk, Poland

**Keywords:** reprogramming, wisent, pluripotency factors, resistance

## Abstract

The European wisent (*Bison bonasus*), an iconic yet genetically vulnerable species, faces ongoing conservation challenges due to a restricted gene pool. Advances in induced pluripotent stem cell (iPSC) technology offer promising prospects for preserving and restoring genetic diversity in endangered species. In this study, we sought to reprogram wisent somatic cells into iPSCs using the PiggyBac transposon system, a non-viral method known for being successfully applied in bovine species. We applied a six-factor reprogramming cocktail (*OCT4*, *SOX2*, *KLF4*, *LIN28*, *c-MYC*, *NANOG*) alongside small-molecule enhancers to fibroblasts isolated from adult wisent tissue. While initial colony formation was observed, the reprogrammed cells exhibited limited proliferation and failed to maintain stable pluripotency, suggesting intrinsic barriers to complete reprogramming. Despite optimizing culture conditions, including hypoxia and extracellular matrix modifications, the reprogramming efficiency remained low. Our findings indicate that wisent somatic cells may require alternative reprogramming strategies, such as new-generation delivery systems and epigenetic modulators, to achieve stable iPSC lines. This study underscores the need for species-specific optimization of reprogramming protocols and highlights the potential of emerging cellular technologies for conservation efforts. Future research integrating advanced reprogramming tools may pave the way for genetic rescue strategies in wisent and other endangered species.

## 1. Introduction

The development of modern cellular technologies based on recent findings in stem cell research holds great promise for reversing biodiversity loss. “Conservation by cellular technologies” is a term that has recently appeared in the context of a rescue plan for the last northern white rhinoceros [1]. It is based on recent advances in embryology, biology of reproduction, and stem cell research—more specifically, embryo-derived (hereafter called embryonic stem cells, ESCs) and induced pluripotent stem cells (iPSCs) [2]. The promise of these cells reaches beyond their applications in regenerative medicine as the knowledge and technology can be expanded for the diversity of species under assault.

Many continued threats still exist for the reconstructed wisent populations in Europe, including limited natural habitat, the pressure of tourism, a broad spectrum of diseases, and varying genetic purity that is manifested by the existence of interspecies hybrids [3]. The extraordinary measures are necessary to prevent the risk of inbred and extinction. To this aim, the repository of cells and germplasm collected from many individuals can ensure the preservation of the existing gene pools [4,5]. Fibroblasts from living and deceased animal tissue can be obtained from simple and non-invasive skin biopsy and can be banked. However, these cells’ potential is restricted by their limited growth capacity in vitro. Furthermore, once preserved, the gene pool should be reintroduced into existing populations. Derivation of ESCs from early preimplantation embryos of wisent subspecies would be the golden standard and method of choice for providing both highly proliferative cells and the possibility of gene transfer through germline in the next generations. However, in the case of large, free-roaming animals, embryo recovery is beyond feasible, posing significant ethical and technical difficulties.

The above-mentioned shortcomings can be easily overcome by deriving iPSCs, sharing key ESCs properties, and obtaining by cellular reprogramming of already existing and banked wisent somatic cells.

The technology of cell reprogramming has been widely used since 2007, when both Yamanaka’s and Thomson’s teams reported the reprogramming of somatic cells to a pluripotent state through the ectopic expression of four transcription factors, *OCT4*, *SOX2*, *KLF4*, and *c-MYC* [6] and *OCT4*, *SOX2*, *NANOG*, and *LIN28* [7]. The resulting cells were designated iPSCs to distinguish them from the embryo-derived ESCs. Both ESCs and iPSCs can self-renew and are pluripotent, but more importantly, they can contribute to the development of the embryo proper. Combining these features with the advancing reproductive sciences may enable the future reintroduction of lost genotypes into the wisent population.

Generation of iPSCs has been successfully extended to both animals of agricultural interest [8,9] and some rare and nearly extinct species like rhinoceros, drill, snow leopard [10,11,12], or more recently, giant panda [13]. Efficient generation of bovine iPSC with the use of both viral vectors [14] and non-viral methods [15] has been described, together with a description of conditions for obtaining cells showing high pluripotency status [16].

Cell reprogramming that relay on viral gene delivery of reprogramming factors is associated with considerable risk of genotoxicity, insertional mutagenesis, and further tumorigenesis. In contrast, the PiggyBac transposon system offers several advantages: it enables robust expression of reprogramming factors from a single polycistronic cassette, accommodates large DNA fragments, and allows for footprint-free excision of the vector from the host genome [17]. These features make PiggyBac particularly appealing for conservation-related applications, where transgene-free cell lines are highly desirable.

In light of the above, we aimed to apply the piggyBac transposon system that has been described as successful for the derivation of iPSCs from various species, including cattle [15,18,19], to derive iPSCs from somatic cells of wisent.

## 2. Results

### 2.1. Reprogramming Factors Delivery to Wisent Somatic Cells

At first, a repository of wisent somatic cells (ear fibroblasts) was established in early 2000 at the Institute of Genetics and Animal Biotechnology and was tested for after-thawing survival, proliferation, and mycoplasma contamination. Based on the mycoplasma test, morphology, and proliferation rate, we have selected two male primary adult fibroblast (BAF) cell lines. In addition, wisent ovarian granulosa cells (BOGC) and ovarian fibrocytes (BOF) have been obtained from the Warsaw University of Life Sciences.

Next, the establishment of reprogramming factors delivery parameters, namely transposon pPB-OSKLMN and transposase ratio, total DNA amount, and time of lipofection to BAFs, BOFs, and BOGCs, has been performed. Successful lipofection and transposition were determined in the somatic cells by transposase red fluorescence and *OCT4* transgene immunostaining 48–72 h after lipofection.

**Figure 1:**
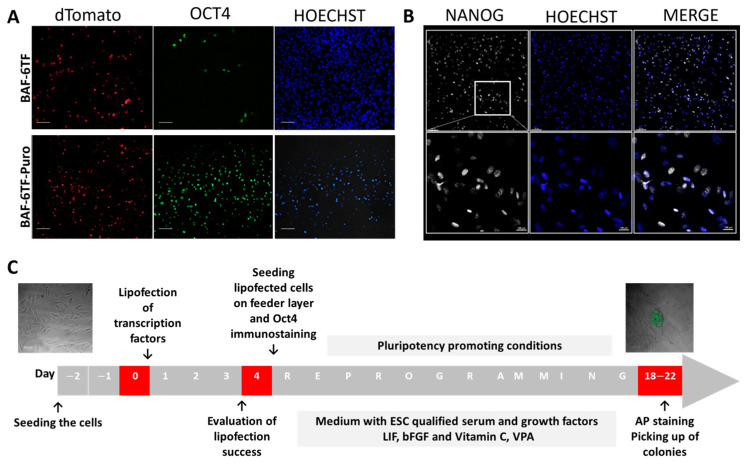
(**A**) Wisent (*Bison bonasus*) adult fibroblast (BAF) after lipofection carrying six reprogramming factors (BAF-6TF) and BAF carrying six reprogramming factors after puromycin selection (BAF-6TF-Puro) characteristics. Live naïve fluorescence of dTomato for transient transposase expression 48 h post-lipofection and immunostaining for *OCT4* after puromycin selection, with Hoechst counter staining for nuclear DNA; scale bar 70 μm (**B**) Immunofluorescent staining for *NANOG* in BAF-6TF-Puro cells with Hoechst counter staining for nuclear DNA; scale bar 100 μm (**C**) Experimental outline.

For all somatic cells lipofected with 700–1000 ng of total DNA and the molar ratio 1:4 of transposase (Tp) and transposon pPB-OSKMLN, the number of red fluorescent cells and *OCT4*-positive cells was detected, albeit at a low percentage (Figure 1A, BAF-6TF). Seeing a relatively small percentage of *OCT4*-positive cells after lipofection, we have decided to switch to a transposon vector for one carrying the puromycin resistance gene, pPB-OSKLMN-PURO, to select the population that would homogenously express reprogramming genes. The lipofection was repeated, and puromycin selection was conducted for 7 days while only cells carrying the puromycin resistance cassette were amplified, thus expressing all six factors. Due to their fragility, restricted life span, and difficulty in handling ovarian cells, only wisent skin fibroblasts carrying six transcription factors (6TFs) were used for subsequent studies (BAF-6TF). We have confirmed that transcription factors: *OCT4*, *SOX2*, *c-MYC*, *KLF4*, and *NANOG* were expressed in the amplified wisent adult fibroblasts after puromycin selection. Both *c-MYC* and *KLF4* were also expressed in non-lipofected cells showing the typical pattern of expression for skin dermal fibroblasts [20,21]. Additionally, we have performed the immunostaining of fibroblasts for *OCT4* and *NANOG* (Figure 1A,B), revealing that both proteins were detected in virtually all cells, albeit at different levels.

### 2.2. iPSC Derivation in Established Conditions

**Figure 2:**
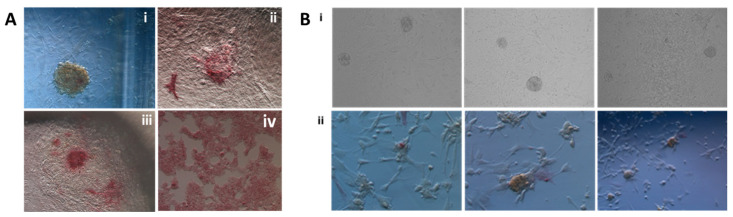
(**A**) Alkaline phosphatase (AP) staining of colonies appearing during reprogramming of BAF-6TF-Puro cells (day 21) with colonies showing weak (yellow) AP staining with residua AP + violet cells (i), partially AP-positive colony (ii) and AP- positive colony (iii) and control staining of mouse ESC (iv). (**B**) Reprogramming of Wisent (*Bison bonasus*) adult fibroblast carrying six reprogramming factors after puromycin selection (BAF-6TF-Puro). (i) Bright field colonies appearing during reprogramming of BAF-6TF-Puro on the laminin-521 substrate (day 7). (ii) Alkaline phosphatase staining of passaged colonies (passage 1).

Having in mind the plethora of protocols that are available and have been used for iPSCs derivation in different species, including cattle, we sought to apply the well-described protocol for the derivation of iPSCs from cattle fetal fibroblasts with the transposon system [15]. We have applied conditions that led to the derivation of stable bovine iPSCs, namely the growth on an inactivated layer of feeder cells (mouse embryonic fibroblasts, MEF) in the medium supplemented with fetal bovine serum (FBS) that supports the development of embryonic stem cells and both LIF and bFGF (Figure 2A). BAF-6TF wisent fibroblasts were seeded and grown for 21–24 days in those conditions. The first colonies demonstrating typical morphology of iPSCs were spotted after 10–14 days of exposition to pluripotency-promoting factors. Specific phenotypic assessment of pluripotent stem cell colonies with alkaline phosphatase (AP) staining at day 22 revealed that most of the colonies showed weak AP expression (yellow colonies), some were partially AP-positive and only a few were regular and doomed-shaped, opaque, and showing violet AP staining (Figure 2B). The colonies were small in number, and the estimated colony formation rate on day 22 was 0.04 (counted as the total number of appearing colonies from all seeded cells).

Knowing that fibroblasts derived from aged animals can exhibit difficulty in reprogramming due to epigenetic roadblocks, we have attempted to increase the ratio of colony formation by adding to the culture medium two enhancing reprogramming molecules: valproic acid (VPA) and ascorbic acid (AA) [22,23,24,25]. After 22 days of reprogramming, the total number of colonies slightly increased and estimated colony formation was 0.048. After live alkaline phosphatase stain, the positive colonies were picked and reseeded on laminin 521 or inactivated MEF feeder layer. None of the conditions allowed for the amplification of picked colonies, and they ceased growing after 2 passages.

### 2.3. Determination of Permissible Culture Conditions for iPSCs Derivation

**Figure 3:**
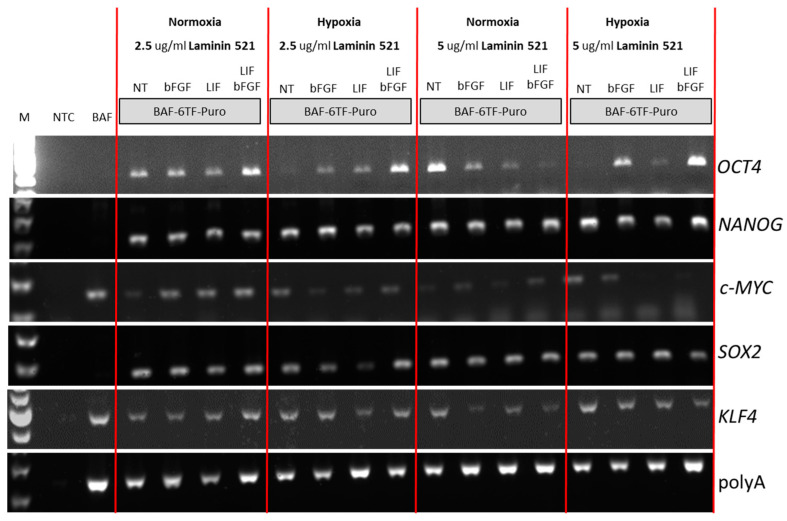
Expression of exogenous reprogramming factors in normoxic and hypoxic conditions on laminin-521 substrate. M—molecular size marker, NTC—no template control, Nt—not treated, BAF-6TF-Puro—Wisent (*Bison bonasus*) adult fibroblast carrying six reprogramming factors after puromycin selection.

As the derivation of iPSCs in previously established reprogramming conditions for bovine fibroblasts resulted in poor outcomes when wisent cells were reprogrammed, we sought to determine more permissive conditions. We have concentrated on two additional elements of the reprogramming niche: surface and oxygen concentration in the growth environment. BAF-6TF cells were grown on laminin 521 [26,27] coated dishes in basal iPSCs medium conditioned on mitotically inactivated MEF feeder, supplemented growth factors combination: bFGF only, LIF only, LIF and bFGF. Cells were grown in hypoxic and normoxic conditions (5% vs. 21% O_2_ in a gas environment). During reprogramming, some fibroblasts created a feeder layer and a few formed colonies. Appearing after 7 days, colonies were small but regular in shape (Figure 2B). Wisent cells cultured in basal media supplemented with bFGF and LIF give rise to at least 2 colonies per cm2 in hypoxia and atmospheric oxygen tension with a colony formation ratio of 0.066. After 14 days, the whole cultures were collected and analyzed by RT-PCR to determine the expression of exogenous pluripotency factors (Figure 3). Albeit exogenous expression of all factors is driven from a single promoter, there are notable differences in their expression pattern. *NANOG*, *SOX2*, and *KLF4* expression are relatively stable in all conditions. High fluctuations, however, are observed for the expression of *OCT4* and *c-MYC*. The downregulation of *OCT4* mRNA is notable when cells are grown in hypoxic conditions on the laminin-521 surface, independent of the laminin-521 concentration. The concentration of laminin in both normoxic and hypoxic conditions has the highest influence on *OCT4* expression when cells are grown in an essential iPSC medium supplemented with both growth factors, bFGF and LIF. However, it is clear that none of the tested conditions is suitable for high exogenous expression of all transcription factors altogether, although the culture in normoxia on 2.5 μg/mL Laminin 521 in the presence of both LIF and bFGF stabilizes (or is most stabilizing for) the expression of exogenous factors.

## 3. Discussion

Although typical symptoms of deep inbreeding depression (observed as reduced fertility and calving performance) have not been noticed in the wisent Lowland line (*Bison bonasus* bonasus), the low genetic variability of the small isolated populations can have serious consequences in the future. The animal of interest, *Bison bonasus*, is closely related to bovine species, as they belong to the same Bovidae family, a subfamily of the Bovinae and Bovini tribe, consisting of genera Bison, Bubalis, and Bos. Thus, knowing the similar genetic background, underlined by the fact that both wisent and cattle may breed, giving viable offspring (żubroń), it was assumed that overexpression of key pluripotency factors, described as efficient in generating iPSCs in cattle [15] also in wisent would lead to the derivation of iPSCs.

Several reprogramming systems and factors have been described for the derivation of iPSCs in cattle [15,16,28,29,30]. To mitigate the risks of viral gene delivery, in our attempt to derive wisent iPSC, we employed a system based on DNA transposon delivery of reprogramming factors [17,31]. The advantages of this system include the possibility of a relatively simple introduction of naked DNA via lipofection or electroporation, host-factor independence, and most importantly, obtaining the iPSCs line without alteration in the genome, as the transiently expressed transposase could remove the integrated transposon. Moreover, PiggyBac transposon vector carrying six transcription factors has been used for successful generation of iPSC cells from fetal bovine fibroblasts, adult baboon fibroblasts, and marmoset post-natal fibroblasts, making it an ideal choice for our study [15,18,19,32].

Additionally, we have opted for the usage of 6 reprogramming factors (OSKLMN) instead of the more common 4 as there was evidence that 6 factors give a higher reprogramming percentage in bovine fetal fibroblasts [15]. Despite these promising methods, the transposition of six transcription factors appeared insufficient for establishing bona fide iPS cell lines from wisent adult fibroblasts. This came as an unforeseen result as the same method, vectors, and conditions were compatible with the reprogramming of both bovine, baboon, and marmoset primary fetal fibroblasts [15,18,19]. Albeit low efficiency, putative iPSCs colonies appeared in culture after three weeks of exposition of wisent somatic cells to pluripotency promoting condition. However, after the formation of colonies, the proliferation of cells was not sustained, regardless of the applied boosting conditions. Applying epigenetic modifiers like VPA and AA [22,23,24,25] or culture niche modifications like hypoxia [33,34,35], and extracellular matrix alteration [26,27], which were proven to enhance the reprogramming process, appeared to be insufficient to overcome the block for sustained and stable pluripotency. These indicate that induced pluripotency was not maintained and applied conditions were insufficient for prolonged self-renewal. Such resistance and lack of sustained self-renewal have also been observed by others when attempting to reprogram bovine fetal somatic cells using a retroviral expression system and five reprogramming factors [28]. Limited propagation of putative bovine iPSCs has also been reported by others [36,37].

A fundamental and mandatory factor in the success of reprogramming is the high and stable expression of exogenous reprogramming factors. The population of wisent primary fibroblasts used here is heterogeneous in nature and not clonal, composed of cells in different proliferative states, often quiescent [38]. Additionally, cells after lipofection of reprogramming factors and selection showed a heterogeneous pattern of expression of *NANOG* in the starting population, most probably as a result of different events of polycistronic cassette integrations into the genome of each cell. Thus, each cell in the heterologous population expresses TFs at different levels. Moreover, observed exogenous transcription factor expression fluctuations in various reprogramming conditions generate further hurdles for successful reprogramming.

The reprogramming outcome is influenced by the method, culture conditions, or the presence of small molecules, and the cell type, donor age, and resulting epigenetic landscape. A high number of scientific reports described the successful derivation of iPSC from various somatic cell types in cattle like Wharton cells, embryonic fibrocytes, mesenchymal stem cells, and testicular cells [15,28,30,39]. However, virtually none describes the derivation of iPSCs from adult and aged somatic fibroblasts. Fibroblasts used in the presented study, obtained from deceased animals, chosen for selective killings because of the disease and age, most probably accumulated during the life span the significant number of mutations and epigenetic marks [40,41] that are impossible to be erased by forced expression of six pluripotency factors, small enhancing molecules or hypoxia. Moreover, low genetic variability in genetically homogenous wisent populations per se [42] reflects epigenetic rigidity that can represent a barrier and effect in reduced efficiency or even a roadblock in the reprogramming process. In such a setting, epigenetic marks, such as DNA methylation patterns and histone modifications, are more difficult to erase and reset, even after applying histone deacetylase HDAC inhibitors, like VPA and AA.

The selection of aged somatic cells for reprogramming is a limitation of the presented report. Using fetal wisent fibroblasts or adult blood cells (CD34+) would improve reprogramming efficiency, however due to ethical, logistical, and regulatory constraints, sample collection is highly restricted and depends on opportunistic access. Future studies should investigate the molecular barriers to reprogramming in wisent cells. Single-cell RNA sequencing could uncover transcriptional heterogeneity and identify cells with incomplete activation of the pluripotency network in response to reprogramming conditions. DNA methylation profiling and analysis of repressive histone marks may reveal epigenetic roadblocks that persist during reprogramming [43]. The presence of DNA methylation and trimethylation of H3K9 inherited from somatic cells is known as epigenetic barrier for reprogramming during iPSC induction [44,45]. These factors are especially relevant for the wisent, a species with very low genetic diversity due to a severe bottleneck and reconstitution from a small founder population. Integrating these molecular approaches in future work would help clarify whether the barriers are due to cell-intrinsic epigenetic features or broader species-specific limitations, ultimately guiding strategies for iPSC derivation in endangered species.

In parallel, applying alternative reprogramming methods such as Sendai virus, episomal vector, or mRNA-based systems would help clarify whether the observed difficulties in deriving wisent iPS cells are specific to the piggyBac delivery system or reflect fundamental biological characteristics of wisent aged somatic cells.

Most scientific approaches toward understanding pluripotency acquisition and maintenance in Bovidae are based on mouse and human models. The restrictions of these models are numerous, the most important being that it is impossible to extrapolate knowledge and know-how to other species. This is mirrored by long-time difficulty and many failures in establishing bona fide naïve embryonic stem cells from cattle [46,47].

Some approaches have been proposed to overcome the roadblocks of reprogramming bovine iPSCs. The overexpression of SV40 large T antigen oncogene together with six pluripotency TF combined with several enhancers [29] and lysine-specific demethylase 4A (KDM4A) and the other reprogramming factors *OCT4*, *SOX2*, *KLF4*, *c-MYC*, *LIN28*, and *NANOG* in bovine mesenchymal stem cells [30] led to the derivation of iPSCs with higher efficiency and better characteristics. The examples mentioned above underline the necessity of bovine cells, regardless of their origin, to receive an additional enhancement to acquire more accessible pluripotency states attained in mouse or human cells.

Similarly, a recent effort to derive iPSC from the Giant Panda came to fruition only after the application of several inhibitors of the ALK5 pathway and an additional targeting number of signaling pathways, including TGFβ, Wnt, and others to induce and maintain primed pluripotency state [13].

The picture emerging from reported data here is that regardless of conditions that have been applied, the reprogramming of wisent adult fibroblasts is stuck after colony formation. Although the initial reprogramming step led to the formation of AP-positive colonies, indicating that induction of pluripotency has been achieved, the maintenance was unsuccessful. Encountered limitations came from the limited number of individuals available for the study and the limited choice of cells, which led to bias in the results. However, we believe that the challenges commonly encountered by the scientific community in endangered species preservation are not sufficiently reported or highlighted.

Thus, the unique biology of bovine cells and the need for more species-specific protocols to overcome the complexities of iPSCs derivation in Bovidae, including wisent and American bison, demands further research to unlock the full potential of iPSCs for genetic rescue and biodiversity conservation for threatened populations and species.

## 4. Materials and Methods

### 4.1. Cell Source

#### 4.1.1. Wisent Adult Fibroblasts (*Bison bonasus* Adult Fibroblasts—BAF)

Two male and one female fibroblasts, derived from skin biopsy samples collected post-mortem from individuals chosen for selective culling in early 2003 at Białowieża Forest and stored in a cell bank at the Institute for Genetics and Animal Biotechnology, were used [48].

Fibroblasts of early passage (passages 2–4), confirmed as negative for Mycoplasma infection by PCR testing (VenorGeM Mycoplasma Detection Kit, Minerva Biolabs, Berlin, Germany) were subsequently used for experimental purposes.

#### 4.1.2. Wisent Adult Ovarian Granulosa Cells (*Bison bonasus* Adult Ovarian Granulosa Cells—BOGCs) and Ovarian Fibroblasts (*Bison bonasus* Adult Ovarian Fibroblasts—BOFs)

Wisent ovarian granulosa cells and ovarian fibrocytes have been obtained from the Warsaw University of Life Sciences. Briefly, 10 female wisents, 3–11 years old, from different herds in Poland, were culled out of the reproductive season (October–March) for reasons other than infertility. Each collected ovary was used for the isolation of oocytes, granulosa cells, and ovarian fibrocytes according to Duszewska et al. protocols [4]. Ovarian granulosa cells were aspirated after cumulus-oocyte complexes (COCs) isolation from ovarian follicles, whereas ovarian fibrocytes were recovered after COCs and granulosa cell isolation. Welfare regulations were approved by the Local Ethics Commission for Animal Experiments of Warsaw University of Life Sciences. 

### 4.2. Cells and Culture Conditions

Wisent Adult Fibroblasts (*Bison bonasus* Adult Fibroblasts—BAFs), Wisent Ovarian Granulosa Cells (*Bison bonasus* Ovarian Granulosa Cells—BOGCs), and Wisent Ovarian Fibroblasts (*Bison bonasus* Ovarian Fibroblasts—BOFs) were cultured in a humidified atmosphere at 37.5 °C, 5% CO_2_. Media used for the culture were Dulbecco’s Modified Eagle Medium (DMEM, Gibco, Grand Island, NY, USA) and Dulbecco’s Modified Eagle Medium: Nutrient Mixture F-12 (DMEM/F12, Gibco, Grand Island, NY, USA), with 10% Fetal Bovine Serum (FBS, Gibco, Grand Island, NY, USA), 1x Penicillin/Streptomycin/Glutamine (PSG, Gibco, Grand Island, NY, USA), 1% Non-Essential Amino Acids (NEAA, Gibco, Grand Island, NY, USA). All cells used in all the experiments were between passages 2–6.

### 4.3. Lipofection

Two PiggyBac transposon plasmids carrying reprogramming factors were used in this research for lipofection: (i) pTT-PB-Pcag-SOKMLN [15], carrying a cassette of six pluripotency-associated human transcription factors *POU5F1 (OCT4)*, *SOX2*, *LIN28*, *NANOG*, *c-MYC*, and *KLF4* separated by self-cleaving 2A peptide, under control of single CAGGS promoter and flanked by ITRs and (ii) pTT-PB-Pcag-SOKMLN-Puro, additionally carrying a sequence for a puromycin resistance gene under the control of a separate promoter in a reverse orientation [17,18]. The transposase helper plasmid, pcA3-PBase-IRES-tdTomato, has a hyperactive transposase gene driven by CMV promoter and IRES sequence followed by a protein of red fluorescence-tdTomato [15].

For lipofection, cells were seeded at 5–10 × 10^3^/cm^2^ and transfected with a plasmid mix of transposon and transposase in a total DNA amount of 700–1000 ng using Lipofectamine LTX (Thermo Fisher Scientific, Waltham, MA, USA) according to the manufacturer’s suggestions for fibroblast cells.

### 4.4. Alkaline Phosphatase (AP) Staining

Alkaline phosphatase live stain has been performed with Alkaline Phosphatase Live Stain (Invitrogen, Carlsbad, CA, USA) according to the manufacturer’s instructions. In brief, after growth medium removal and washes with serum-free medium (SFM), cells were incubated with 1x working solution for 45 min at 37 °C, 5% CO_2_. After incubation cells were washed with serum-free medium twice for 5 min to remove the excess AP live stain. After the final wash, SFM was added, and cells were observed under the fluorescent microscope Zeiss Axiovert 200 M.

AP staining of fixed colonies started with fixing the cells with 4% Paraformaldehyde (PFA) for 10 min at room temperature. After that time, PFA was carefully discarded, and cells were washed 5 times with DPBS. The AP staining was performed with a Leukocyte Alkaline Phosphatase Kit (Sigma-Aldrich, St. Louis, MO, USA) based on manufacturer instructions. Briefly, for staining solution preparation, 125 μL of Sodium Nitrate and 125 μL of FRV-Alkaline Solution were mixed and incubated for 2 min at room temperature, then transferred to 5.6 mL of distilled water, and following 125 μL of Naphtol AS-BI Alkaline Solution was added. Fixed cells were incubated with a prepared staining solution for 30 min at 37 °C, 5% CO_2_, and protected from the light. After the incubation time, the dishes were washed three times with DPBS, and colonies were observed under the microscope (Nikon Elipse TE300, Nikon Corporation, Tokyo, Japan).

### 4.5. Immunostaining

Lipofected cells, after RFP fluorescence evaluation, were fixed for 10 min using 4% PFA, permeabilized with 0.5% Tween in PBS, and blocked with 10% normal donkey serum for 30 min. Afterward, cells were washed three times with DPBS. Primary antibody was applied overnight, both anti-*OCT4* (sc-8629 c-20, Santa Cruz, CA, USA) and anti-*NANOG* (500-P236 Peprotech) in 1:1000 dilution in 1% Donkey Serum (DS). The plate was left overnight at 4 °C. The next day, the primary antibody was discarded, and cells were washed three times for 6 min at the mixing block. Alexa Fluor 488 or 647 conjugated in 1:1000 dilution, prepared in 1% DS, was used as the secondary antibody. Incubation lasted for 30 min at 37 °C. After the incubation time, cells were washed three times quickly and three times for 10 min at the mixing block. Hoechst stain was used for nucleus staining (1:1000 dilution in DPBS) for 10 min in the dark at room temperature. After 10 min, the cells were washed three times with DPBS and observed under the confocal microscope (Nikon A1R, Nikon Corporation, Tokyo, Japan).

### 4.6. PCR

RNA was isolated from frozen pellets using QIAamp RNA Blood Mini Kit (Qiagen). A reverse transcription reaction with 100–500 ng RNA was performed following the manufacturer’s protocol (QuantiTect Reverse Transcription Kit, Qiagen, Venlo, The Netherlands). The cDNA samples were subjected to Polymerase Chain Reaction (PCR) amplification with the primer pairs listed in Table 1. PCR was done using DreamTaq DNA Polymerase (Thermo Fisher Scientific, Waltham, MA, USA) in the presence of 1% DMSO, 10 μM of each primer, 0.1 U Taq polymerase, and 1x reaction buffer containing 2 mM MgCl2 and 0.4 μM dNTPs. PCR was done using a thermal cycle as follows: initial denaturation 95 °C for 1 min, denaturation 95 °C for 30 s, annealing 56 °C for 30 s, elongation 72 °C for 1 min, and final elongation 72 °C for 5 min. Then, PCR products were separated in 2% agarose gel.

### 4.7. Culture Conditions for iPSCs Derivation

Lipofected cells (200 cells/cm^2^) were seeded on a feeder layer of mitomycin-C (MMC) treated mouse embryonic fibroblast (MEF, CF1 strain, Sigma-Aldrich, St. Louis, MO, USA) or on rhLaminin-521 coated dishes. MEFs were treated with MMC of final concentration 5–10 μg/mL for 2–3 h, then trypsinized (Gibco, Grand Island, NY, USA) and seeded at the density of 2.81 × 10^4^ per cm^2^. Dishes were coated with rhLaminin-521 (0.5 μg/cm^2^) for 2 h at 37 °C, 5% CO_2_. Cells were grown at 37 °C, 5% CO_2_ (Normoxia) and 5% CO_2_, 5% O_2_ and (Hypoxia). The basal iPSCs medium was composed of Dulbecco’s Modified Eagle Medium: Nutrient Mixture F-12 (DMEM/F12, Gibco, Grand Island, NY, USA), supplemented with 20% FBS (ESC-grade), 1X Penicillin/Streptomycin (P/S, Gibco, Grand Island, NY, USA), 1% non-essential amino acids (NEAA), leukemia inhibitory factor (LIF, Sigma-Aldrich, St. Louis, MO, USA) 1000 IU/mL and thermostable basic fibroblast growth factor (bFGF, Thermo Fisher Scientific, Waltham, MA, USA) 20 ng/mL were added from the beginning of the derivation. From day 4 until day 14, valproic acid (VPA, sodium salt, Sigma-Aldrich, St. Louis, MO, USA) 0.5 mM was introduced. Ascorbic acid (AA, Sigma-Aldrich, St. Louis, MO, USA) was added when stated for the whole duration of reprogramming at 25 μg/mL. The medium was changed daily, starting from day 2 after cell seeding on the mitotically inactivated MEF feeder. The colonies were picked manually, with a fire-pulled glass pipet, aspirated, and dissociated with TripLe (Gibco, Grand Island, NY, USA) dissociation reagent for 5 min. Next, the cells were centrifuged and seeded on freshly prepared mitotically inactivated MEFs feeder or rhLaminin-521 coated surface.

## 5. Conclusions

Using the piggyBac transposon system, we attempted to reprogram wisent (*Bison bonasus*) somatic cells into iPSCs. Despite applying six reprogramming factors and enhancing small molecules, we encountered significant barriers, with colonies failing to maintain long-term pluripotency. While we utilized state-of-the-art methodologies available at the time, our results suggest that wisent adult fibroblasts require additional support to maintain the induced reprogramming state. Recent advances, including improved reprogramming systems, alternative delivery methods, and refined chemical inhibitors, may enhance efficiency and stability in future attempts.

Our findings highlight the need for species-specific optimization of iPSC protocols, particularly for endangered species. By integrating new-generation reprogramming tools and epigenetic modulators, future research may unlock the potential of iPSCs for genetic rescue and conservation efforts.

## Figures and Tables

**Table 1:** Sequences, annealing temperature, and product size of primers.

Gene/Target	Forward Primer	Reverse Primer	Product Size (bp)	Annealing Temperature	Reference
*POU5F1 (OCT4)*	GTTCTCTTTGGAAAGGTGTTC	ACACTCGGACCACGTCTTTC	313	56 °C	[15]
*NANOG*	AAACAACTGGCCGAGGAATA	AGGAGTGGTTGCTCCAAGAC	194	56 °C	[49]
*SOX2*	CACAACTCGGAGATCAGCAA	CATGAGCGTCTTGGTTTTCC	162	56 °C	[49]
*c-MYC*	TGGACGCTAGATTTCCTTCG	GCTGCTGCTGGTGGTAGAAG	155	56 °C	[49]
*KLF4*	GCCCCTAGAGGCCCACTT	CACAACATCCCAGTCACAG	433	56 °C	[15]
*polyA*	GTTTCCTCGGTGGTGTTTCCTGGGCTATGC	TGGAGTTCTGTTGTGGGTATGCTGGTGTAA	252	56 °C	[50]

## Data Availability

The raw data supporting the conclusions of this article will be made available by the authors on request.

## Abbreviations

The following abbreviations are used in this manuscript:

6TFs	Six transcription factors
AA	Ascorbic acid
AP	Alkaline phosphatase
BAFs	*Bison bonasus* adult fibroblasts
BOFs	*Bison bonasus* Ovarian Fibroblasts
BOGCs	*Bison bonasus* Ovarian Granulosa Cells
COCs	Cumulus-oocyte complexes
ESCs	Embryonic stem cells
FVBS	Fetal bovine serum
iPSCs	Induced pluripotent stem cells
MEF	Mouse embryonic fibroblasts
VPA	Valproic acid
